# Antipsychotic Drug Fluphenazine against Human Cancer Cells

**DOI:** 10.3390/biom12101360

**Published:** 2022-09-23

**Authors:** Diana Duarte, Nuno Vale

**Affiliations:** 1 OncoPharma Research Group, Center for Health Technology and Services Research (CINTESIS), Rua Doutor Plácido da Costa, s/n, 4200-450 Porto, Portugal; 2 Faculty of Pharmacy, University of Porto, Rua Jorge Viterbo Ferreira, 228, 4050-313 Porto, Portugal; 3 CINTESIS@RISE, Faculty of Medicine, University of Porto, Alameda Professor Hernâni Monteiro, 4200-319 Porto, Portugal; 4 Department of Community Medicine, Health Information and Decision (MEDCIDS), Faculty of Medicine, University of Porto, Rua Doutor Plácido da Costa, s/n, 4200-450 Porto, Portugal

**Keywords:** anticancer activity, fluphenazine, antipsychotic drugs, human cancer cell lines

## Abstract

Drug repurposing is a strategy that can speed up and find novel clinical uses for already-approved drugs for several diseases, such as cancer. This process is accelerated compared to the development of new drugs because these compounds have already been tested in clinical trials and data related to their pharmacokinetics is already described, reducing the costs and time associated with the development of new anticancer therapeutics. Several studies suggest that the repurposing of fluphenazine for cancer therapy may be a promising approach, as this drug proved to reduce the viability of diverse cancer cell lines. In this review, intensive research of the literature was performed related to the anticancer potential of fluphenazine in different human cancer cells. We have found several research articles on the cytotoxic effect of fluphenazine in lung, breast, colon, liver, brain, leukemia, oral, ovarian, and skin cancer and have summarized the main findings in this review. Taken together, these findings suggest that fluphenazine may regulate the cell cycle, reduce cell proliferation, and cause apoptosis in several types of cancer cells, besides being an established calmodulin inhibitor. It was also found that this drug is able to target cancer-related proteins, such as ABCB1 and P-glycoprotein as well as to regulate the Akt and Wnt signaling pathways. Some studies also refer this drug causes DNA alterations and interferes with cell invasion and migration ability as well as with ROS generation. Collectively, these results imply that fluphenazine may be a favorable compound for further research in oncologic therapy.

## 1. Introduction

Cancer is one of the most important major health problems around the globe, being the second leading cause of mortality in the United States (US) [1]. The most recent statistics on incidence and mortality are still impacted by the coronavirus disease 2019 (COVID-19) pandemic, due to the reduction of access to health care virtue of lockdowns, and fear of COVID-19 exposure, which resulted in setbacks in diagnosis and therapy [2]. The most recent report on cancer statistics published by the American Cancer Society estimates that in 2022 more than 1,900,000 new cancer cases and more than 600,000 cancer deaths will occur in the US [1]. Lung cancer is expected to cause 350 deaths per day, maintaining the first position as the leading cause of cancer death in both sexes. Breast and colon cancer follow the list as the second and third most diagnosed types of cancer, accounting for 43,780 and 52,580 estimated deaths in the US, respectively. Brain and other nervous system cancers will also account for 25,050 estimated new cases and 18,280 deaths; leukemia with 60,650 new cases and 24,000 deaths; liver and intrahepatic bile duct cancers with 41,260 new cases and 30,520 deaths; lymphoma with 89,010 new cases and 21,170 deaths; pancreatic cancer with 62,210 new cases and 49,830 deaths; and skin cancer (excluding basal and squamous) will account for 108,480 new cases and 11,990 deaths, approximately [1]. These numbers reflect the urgent need for the development of novel and more efficient anticancer treatments that help reduce the mortality of cancer patients and improve their life quality during their treatments.

Drug repurposing (also called drug repositioning) is a strategy that makes use of drugs already approved in the market by the Food and Drug Administration (FDA) for novel therapeutic indications, besides their original ones [3]. Sometimes, it can also expand the use of a certain drug to another similar disease, such as another type of cancer. This strategy takes less time and investment than the development of novel drugs, as the toxicological profiles of the drug are well-defined, reducing the time needed for approval and increasing the chance of these drugs passing into clinical trials [4,5]. Different central nervous system (CNS) drugs have already been explored as repurposed drugs in different studies for the treatment of several diseases and the preliminary results in human cancer cells demonstrate that this class of drugs may possess promising anticancer effects [6,7,8,9,10].

Recently, some reviews described the potential of different CNS drugs such as chlorpromazine, thioridazine, and trifluoperazine in cancer and other diseases, such as Alzheimer’s disease and Parkinson’s disease [11,12,13]. Another study, published in 2021, reviewed the activity of prochlorperazine, fluphenazine, and perphenazine as potential candidates for use in antitumor therapy [14]. In this review, we will exclusively focus on the promising anticancer effects of fluphenazine against different human cancer cells. To perform this research, PubMed was used in June 2022 to investigate English papers using the terms “cancer AND fluphenazine”. In total, 93 records from PubMed were found. All papers from 1971 to date were scanned and 41 were selected for this review. Some studies including animal cancer cells were also selected for this review due to relevant findings or important historical background.

**Figure 1:**
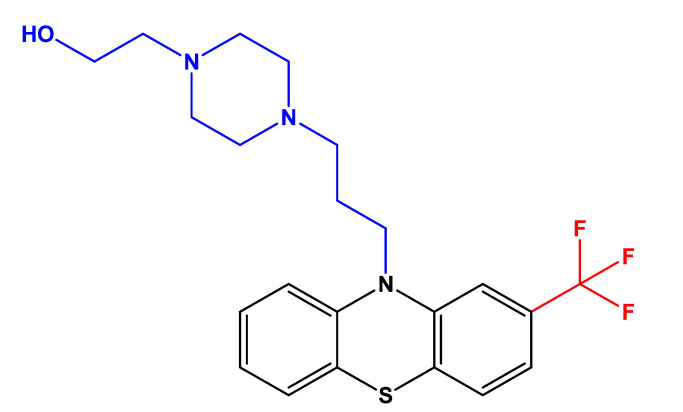
Chemical structure of fluphenazine. This drug consists of a phenothiazine ring system, with a piperazine side chain (represented in blue) attached to the nitrogen in the para-thiazine ring and a trifluoromethyl group (represented in red) attached to the second benzene ring. The structure was obtained using ChemDraw software (version 12.0, PerkinElmer, Inc., Waltham, MA, USA).

Fluphenazine is a drug belonging to the phenothiazines (PTZ) drug class, a group of agents used to treat patients requiring long-term neuroleptic therapy, especially intended for managing schizophrenia and other psychotic disorders [13]. Fluphenazine was included on the World Health Organization (WHO) list of Essential Medicines of 2009 [15]. PTZ are compounds characterized by having a phenothiazine ring system [14]. Specifically, fluphenazine possesses a piperazine side chain attached to the nitrogen in the para-thiazine ring and a trifluoromethyl group attached to the second benzene ring (Figure 1), which increases the efficacy of the drug [14].

Fluphenazine acts on the postsynaptic dopaminergic D1 and D2 receptors in the brain, inhibiting the release of hormones such as dopamine from the hypothalamus and from the hypophysis, which in turn affects different body functions such as basal metabolism, body temperature, wakefulness, vasomotor tone, etc., [16], that can result in CNS side effects during anticancer treatment with this drug. Several studies also suggest fluphenazine induces cell apoptosis, and inhibits mechanisms related to the repair of DNA and other transduction pathways, resulting in damage to the DNA and destabilization of cell membranes [11]. It also inhibits multidrug resistance (MDR) in resistant tumors, inhibits angiogenesis, and presents sedative and antiemetic effects [11]. Fluphenazine is also an inhibitor of calmodulin, the ubiquitous calcium-binding protein [17], which also is responsible for the regulation of different important proteins, having a crucial role in the regulation of cell proliferation, and programmed cell death, autophagy, and cancer [18]. To date, two clinical trials involving the use of fluphenazine in cancer are already available in the ClinicalTrials.gov database: one ongoing phase I clinical trial studying the effect of fluphenazine in relapsed or relapsed-and-refractory multiple myeloma (NCT00821301, [19]) and another phase I/II clinical trial that studied the effect of fluphenazine in treating patients with refractory advanced multiple myeloma (NCT00335647, [20]) completed in 2013, but without publication of the results.

**Figure 2:**
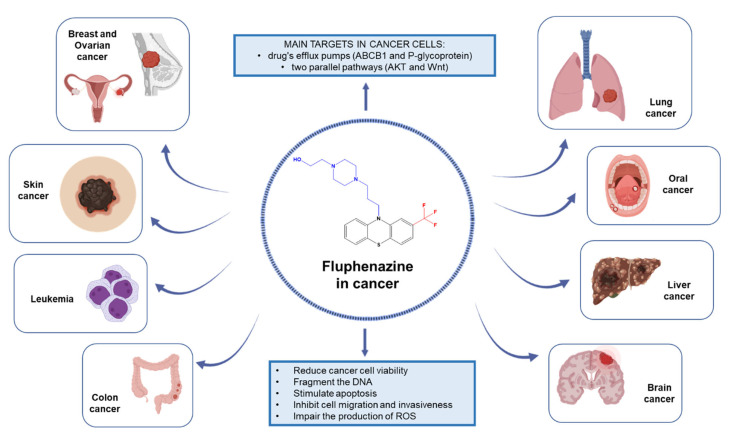
Overview of this review and main findings on the research of fluphenazine in cancer.

Next, we will describe the anticancer effects of fluphenazine against different human cancer cells and will review the promising use of this repurposed drug in the treatment of different types of cancer, as well as its main targets in cancer cells. The overview of this review is described in Figure 2.

## 2. Evidence of Anticancer Effects of Fluphenazine against Different Human Cancer Cells

The first use of fluphenazine in cancer cells dates back to 1971, when Hilf et al. [21] evaluated the effect of fluphenazine on R3230AC mammary carcinoma and mammary glands of the rat, founding for the first time that this drug caused a decrease in the growth of tumor cells, decreasing DNA levels and increasing the levels of free fatty acids and triglycerides, enhancing the activity of enzymes such as glucose-6-phosphate dehydrogenase, NADP-malate dehydrogenase, phosphoglucomutase, and aspartate aminotransferase [21].

Over the years, studies have reported the involvement of fluphenazine in different pathways related to tumorigenesis, and in several important anticancer targets. The calmodulin-inhibiting activity of PTZ was one of the first features to be studied for cancer therapy, in the late 1980s [22], aiming to provide support for the repurposing of this class of drugs in cancer. After that, several studies have demonstrated novel mechanisms of action of these drugs in cancer cells, as next described.

**Figure 3:**
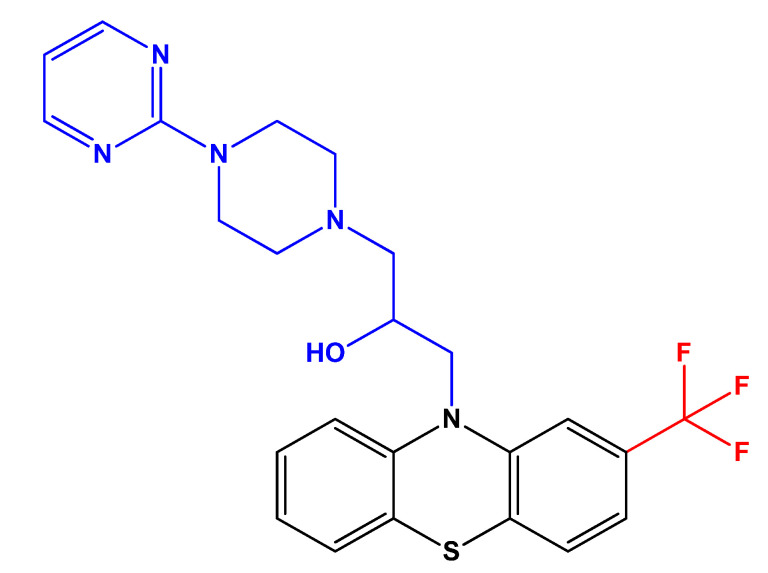
Chemical structure of FPh-prm, an analog of fluphenazine with higher anti-MDR activity. The structure was obtained using ChemDraw software (version 12.0, PerkinElmer, Inc., Waltham, MA, USA). Adapted from [24].

In 2009, Cieslik-Boczula et al. [23], investigated the effect of fluphenazine on the structure of dipalmitoylphosphatidylcholine (DPPC) bilayer. DPPC lipids belong to the phosphatidylcholines lipid group, which are phospholipids present in the structure of cell membranes in eukaryotic cells. Using ATR-IR and ^31^P NMR, the authors found fluphenazine is able to form hydrogen bonds with proton-acceptor carbonyl groups of molecules in DPPC, increasing the fluidization and ultimately causing the destruction of the structure of the lipid bilayer at higher concentrations, which is particularly important for the MDR activity of this drug [23]. A few years later, the same research group evaluated the effects of an analog of fluphenazine with higher anti-MDR activity, FPh-prm (Figure 3), in the structure of the lipid bilayer [24]. The authors have concluded that the formation of domains with distinct content of FPh-prm/DPPC may be associated with an inhibition of the glycoprotein P (P-gp) activity in cancer cells, which decreases the transport of chemotherapeutic drugs to the outside of cell membrane [24].

Another study published by Zhu et al. (2010, [25]) aimed to identify modulators of the Toll-like receptor 3 (TLR3)-IFN regulatory factor 3 (IRF3) signal transduction pathway. TLR3 is an immune sensor of double-stranded RNA and its binding to the RNA activates two important transcription factors: NF-κB and IRF3. The authors found that fluphenazine may modulate and be an inhibitor of this signaling pathway via PI3K signaling pathway, demonstrating an anti-inflammatory role of this drug that may be used in the treatment of some forms of cancer [25].

A study from 2012, published by Jaszczyszyn et al. [26], aimed to evaluate the effect of 17 new derivatives of fluphenazine in the inhibition of P-gp, using genotoxically damaged human lymphocytes. The idea behind the study was based on the established theory that PTZ act as MDR modulators, based on their ability to inhibit P-gp. The authors found that ten of these analogs significantly increased rhodamine 123 accumulation inside cultured lymphocytes and six of them were able to strongly inhibit P-gp activity, being promising candidates, together with fluphenazine, for adjuvant cancer therapies [26].

Another study from Li et al. [27] aimed to identify novel autophagy modulators using a cell-based quantitative high-throughput image screening since reduced autophagy is known to be a marker of tumorigenesis. Using mouse embryonic fibroblasts expressing GFP-light chain 3 (LC3), the authors screened a library of pharmacological compounds both alone and in combination with chloroquine, a well-known inhibitor of lysosomes to find promising autophagic modulators. The authors found that fluphenazine is able to induce autophagy through mTOR inhibition and suggested this drug as a promising candidate for autophagy modulation in cancer [27].

More recently, Bharadwaj et al. (2021, [28]) applied computational screening to find FDA-approved drugs able to modulate Sirtuin 2 (Sirt2) activity. Sirt 2 nicotinamide adenine dinucleotide-dependent deacetylase enzyme has been reported to affect different biological functions and to be involved in several diseases, including cancer, being a potential drug target for cancer therapy. The authors screened and identified 48 drugs with the ability to selectively inhibit this enzyme, including fluphenazine, using molecular docking and dynamics. The developed 3D QSAR models revealed a predicted IC_50_ for this drug of 4.824 µM, supporting the inhibitory potential of this drug as a Sirt2 inhibitor [28].

**Figure 4:**
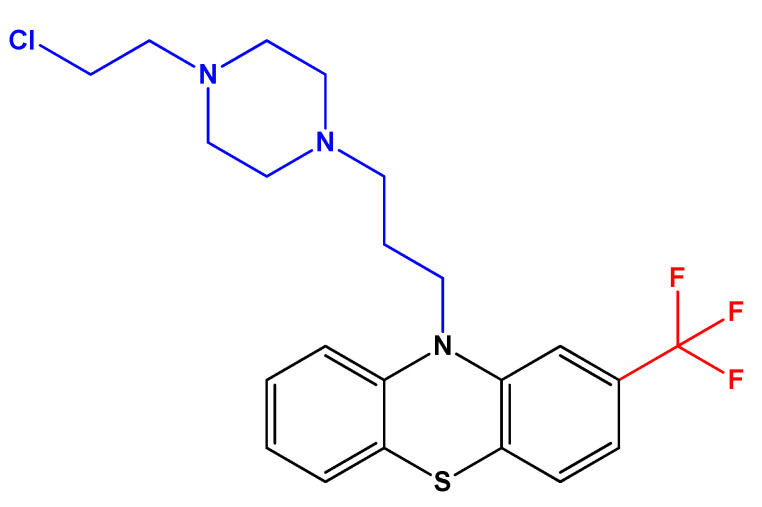
Chemical structure of fluphenazine mustard, an analog of fluphenazine. The structure was obtained using ChemDraw software (version 12.0, PerkinElmer, Inc., Waltham, MA, USA). Adapted from [29].

Furthermore, a recent study aimed to investigate if fluphenazine and its mustard derivative (Figure 4) could synergistically target two cancer-associated targets: calmodulin and the tumor suppressor protein phosphatase 2A (PP2A). These proteins are modulators of the Ras- and MAPK signaling pathways, affecting cell viability and other mechanisms related to cancer cell stemness [29]. The authors used 3D spheroids of different cell lines (breast cancer cells MDA-MB-231 (K-Ras-G13D), bronchioalveolar cancer cells NCI-H358 (K-Ras-G12C), and melanoma cells A375 (B-RAF-V600E)). They found that the combination of fluphenazine (a calmodulin inhibitor) and DT-061 (a PP2A activator) synergistically decreased the formation of spheroids and increased apoptosis in MDA-MB-231 cells, with the mustard derivative showing a more cytotoxic potential [29]. MDA-MB-231 is a triple-negative breast cancer cell line and is representative of one of the most aggressive types of breast cancer. The authors concluded that improved fluphenazine derivatives that retain calmodulin inhibitory and PP2A activating properties, but without neurological side effects, may be promising compounds for anticancer therapy [29].

In the next sections, we will further detail the main findings on the anticancer effect of fluphenazine against different types of cancer cells.

### 2.1. Lung Cancer

In 2009, Hwang et al. [30] evaluated the effect of 180 enzyme inhibitors on tumor necrosis factor-related apoptosis-inducing ligand (TRAIL)-induced apoptosis in the human lung cancer H1299 cell line. TRAIL is responsible for the induction of apoptosis in tumor cells, nevertheless, it is not effective alone in treating TRAIL-resistant tumors. The authors found that fluphenazine-N-2-chloroethane (a fluphenazine derivative and calmodulin antagonist) increases TRAIL-induced apoptosis, by enhancing caspase-8 binding to the Fas-associated death domain (FADD) in the presence of TRAIL. This combination is also able to inhibit AKT phosphorylation, causing a decrease in the expression of anti-apoptotic markers and helping to overcome apoptosis resistance in lung cancers [30].

A study published by Zong et al. in 2011, studied the chemosensitizer effect of different PTZ in different human lung cancer cells. The authors found that fluphenazine is able to increase the sensitivity of U1810 human non-small cell lung carcinoma (NSCLC) cells to the antineoplastic drug cisplatin, commonly used in the management of this type of cancer. These results were further supported by an increase in the expression of caspase-3 (indicative of apoptotic effect) after drug treatment of bleomycin-treated H23 cells, as well as an increase in chemotherapy-induced vacuolation (indicative of lysosomal dysfunction effect), on other cell lines (H23, H125, and U1752) [31].

Another study found that the LC3-II, a marker of autophagy, was also elevated in both small cell lung carcinoma (SCLC) cells and NSCLC treated with fluphenazine [8]. The authors also noted that SCLC cells are more sensitive to this drug than NSCLC cells, after treatment with 10 µM fluphenazine. Nevertheless, the authors also found fluphenazine can induce toxicity in primary fetal lung WI-38 fibroblasts at concentrations above 10 µM, so its use in therapeutical approaches must be carefully evaluated [8].

Another study also evaluated the antiproliferative effect of fluphenazine on different NSCLC cell lines: PC9/R, A549, PC9, H1975, and H522 [32]. The authors found that fluphenazine was cytotoxic to all cell lines at the micromolar range, with PC9/R cells being more sensitive to this drug, with an IC_50_ of 8.08 µM. On the other side, PC9, A549, H522, and H1975 cells demonstrated more drug resistance, with an IC_50_ of 10.90, 58.92, 12.67, and 12.36 μM, respectively [32].

### 2.2. Breast Cancer

Over the years, different studies have demonstrated the potential of fluphenazine against breast cancer cells. A study published in 1989 by Ford et al., demonstrated that different PTZ compounds cause a decrease in the growth of human breast cancer cells and can reverse MDR [33]. MCF-7 cells resistant to the chemotherapeutic drug doxorubicin were treated with fluphenazine and an IC_50_ of 23 µM was obtained. This drug was also demonstrated to be a more potent antagonist of MDR than the other PTZ compounds [33].

More recently, Goyette et al. [34] evaluated the anticancer effects of this drug and other PTZ in two different human breast cancer cell lines: MDA-MB-231 and Hs578T. This study aimed to find if already approved drugs could induce a gene signature similar to that seen with AXL knockdown in triple-negative breast cancer (TNBC) cells. This receptor tyrosine kinase is involved in tumor cell proliferation and several studies suggest its expression is increased in breast cancers with poor prognosis. They found the class of PTZ to be a promising drug class and evaluated the effect of fluphenazine and other PTZ on the proliferation, migration, and invasion of these cells [34]. They found that 20 µM of this compound is enough to reduce MDA-MB-231 and Hs578T cell invasion by 75 and 40%, respectively. Results regarding cell migration also confirmed this drug causes a decrease in the migration of these cancer cells, for both cell lines. Using a luciferase assay, the authors have also found a dose-dependent reduction in the number of proliferating cells, also supported by the results of cell cycle analysis, where cells were arrested in the G1/S phase after drug treatment [34]. Using 3D cancer models, they also found that fluphenazine is able to decrease the formation of tumorspheres, even in concentrations under 20 µM, demonstrating the ability of this drug to decrease tumor progression. Annexin V and propidium iodide analysis demonstrated this drug can induce cell apoptosis and cell death, in both cell lines, especially at higher concentrations. The authors also found this drug reduces PI3K/Akt/mTOR and ERK signaling but does not affect AXL activity. Taken together, these results support the drug repurposing of fluphenazine for the therapy of breast cancer, especially the most resistant ones [34].

Another recent study published by Xu et al. [35] studied the potential of fluphenazine for the treatment of triple-negative breast cancer with brain and lung metastasis, as this type of cancer has a high risk of metastasizing in these organs. They used a wide panel of breast cancer cells including the 4T1, 4T1-Fu5, MDA-MB-231, SKBR-3, MDA-MB-468, BT549, MDA-MB-231-FU10, MCF-7, BT474, and HCC1954 cell lines and had found the IC_50_ of this drug to be under 15 µM for all cell lines, demonstrating a time- and concentration-dependent reduction in the cell viability of 4T1 and MDA-MB-231 cancer cells. They also confirmed this drug was able to cross the blood–brain barrier, with a brain/plasma fluphenazine concentration ratio above 25 for at least 24 h after dosing, indicative of good bioavailability of the drug in the brain. Further studies also demonstrated that low concentrations of fluphenazine resulted in a decrease in the expression of important tumorigenic markers such as p44/42 ERK and phosphorylated AKT [35]. Cell cycle analysis showed that treatments of 24 h with this drug arrest 4T1 and MDA-MB-231 in the G0/G1 phase, supported by a decrease in the expression of cyclin-dependent kinase (CDK) 2, CDK4, cyclin D1, and cyclin E as well as an increased expression of p21 and p27. The study also confirmed this drug is able to induce apoptosis in these cells, using Hoechst 33,342 staining assay and Annexin V-fluorescein isothiocyanate/PI staining. This was further supported by increased levels of cleaved caspase, decreased levels of Bcl-2, and a minor increase of Bax. The authors also found that the apoptotic effect of fluphenazine may be caused by the mitochondrial intrinsic apoptotic pathway, which is also supported by an increase in the generation of reactive oxygen species (ROS) after 12 h of treatment in these cell lines. It was also found that this drug is able to decrease cell migration and invasion [35].

Recently, Duarte et al. developed a new model of drug combination consisting of antineoplastic drugs and different repurposed drugs [36]. Among them, several CNS drugs were studied, including the antipsychotic fluphenazine. MCF-7 cells treated with fluphenazine in increasing concentrations (0.1–100 µM) demonstrated a significant reduction in cell viability in concentrations above 10 µM, with more than 70% cell death. It was also found that the combination of fluphenazine and paclitaxel, a chemotherapeutic compound, resulted in all intermediate concentrations showing a greater anticancer effect than fluphenazine and paclitaxel alone, demonstrating the adjuvant effect that fluphenazine can exert in conventional chemotherapy. The authors also found this combination to be synergic for all concentrations below IC_50_ [36].

The same group also combined fluphenazine with doxorubicin, another anticancer drug, to evaluate if the adjuvant effect of fluphenazine could be appropriate for treatments with other chemotherapeutic agents [37]. It was found that the combination of fluphenazine was not advantageous over each drug alone, both by MTT and SRB assays. Next, the authors evaluated the expression of some epithelial-mesenchymal transition biomarkers in MCF-7 cells treated with paclitaxel + fluphenazine, since this combination resulted in more interesting results. The authors found less β-catenin labeling in the single treatment with fluphenazine. In combination with paclitaxel, there is a lower expression of protein labeling compared to control cells, supporting this drug, both alone and combined, could induce an epithelial-mesenchymal transition reversal [37].

Another study published by Duarte et al. also demonstrated that honeybee venom can synergistically enhance the activity of different CNS drugs, including fluphenazine, in MCF-7 breast cancer cells [38]. The authors treated the cells with fluphenazine alone and combined with increasing concentrations of bee venom and found an IC_50_ of 2.68 µM for a 48 h treatment with fluphenazine. It was also found that the combination of fluphenazine and honeybee venom resulted in better anticancer effects than the combination with doxorubicin, an antineoplastic drug, especially at lower concentrations. These results support the use of fluphenazine in adjuvant strategies for breast cancer.

### 2.3. Colon Cancer

Hypoxic tumor regions are a main feature of solid tumors and refer to sites inside the tumor that lack or have insufficient neo-angiogenesis [39]. This makes it more difficult for chemotherapeutics, radiotherapy, and immunotherapy to reach these regions and consequently contributes to tumor resistance against the therapy. Targeting cells in these regions is, therefore, very important in cancer treatment. A study from 2017 by Klutzny et al. [39] studied the anticancer effect of fluphenazine and other compounds on HCT116 tumor spheroids in hypoxic conditions to find promising compounds that could reach these hypoxic regions. The authors demonstrated this drug is able to reduce 50% of cell viability in 2D cultures of HCT116 cells in concentrations above 10 µM in normoxia, being also capable to inhibit acid sphingomyelinase and consequently cause cellular sphingomyelin accumulation, inducing cell death mediated by the stress-responsive transcription factor ATF4, under hypoxia conditions [39].

Another study from Środa-Pomianek et al. (2018, [40]), evaluated the anticancer effect of fluphenazine and other synthesized derivatives in two human colon adenocarcinoma cell lines: the sensitive (LoVo) and doxorubicin-resistant (LoVo/Dx). These drugs were selected as this class of drugs acts as MDR modulators. The authors found an IC_20_ value of 22 µM and an IC_50_ of 80 µM for both lines treated with fluphenazine, respectively. It was also found that fluphenazine and its derivatives induce apoptosis and autophagy, increase cellular lipid peroxidation, as well as generate ROS. Some fluphenazine derivates were also able to induce more potent anticancer effects in LoVo/Dx cells than in LoVo cells, demonstrating their potential for use in the treatment of resistant cancers [40].

Another study from 2019 [41] evaluated the combination of different PTZ, including fluphenazine, with simvastatin on a resistant LOVO/Dx colon cancer cell line. They studied several aspects of each drug alone and combined and investigated their effect on cell viability and proliferation, cell death, and expression of cyclooxygenase-2 (COX-2). They found that fluphenazine combined with this statin increases doxorubicin cytotoxicity, leading to the accumulation of doxycycline inside tumor cells, decreasing the level of ABCB1 (P-gp) transporter as well as the activity and expression of COX-2 enzyme. The authors also demonstrated this drug is able to influence the expression of different mitochondrial markers such as Bcl-2, Bax, and caspase-3, which can be indicative that this drug induces apoptosis in these multi-drug resistant cells [41].

**Figure 5:**
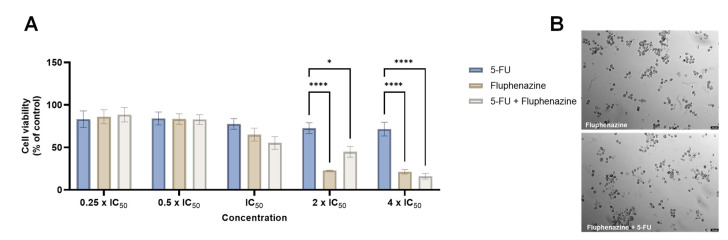
MTT results (**A**) and morphological analysis (**B**) of HT-29 cells treated for 48 h with a combination therapy of 5-FU and fluphenazine. Results are expressed in relation to control cells (treated with vehicle) and represent mean ± SEM. Each experiment was repeated three times (*n* = 3); * statistically significant vs. control at *p* < 0.05. **** Statistically significant vs. control at *p* < 0.0001. Scale bar: 50 µm. Adapted from [34].

A study published by Duarte et al. developed a new model of drug combination consisting of antineoplastic drugs and different repurposed drugs for colon cancer [36]. They included different CNS drugs in the design of the study, including fluphenazine. HT-29 colon cancer cells treated with fluphenazine in increasing concentrations (0.1–100 µM) demonstrated a significant reduction in cell viability in concentrations above 10 µM, with more than 70% cell death and an IC_50_ value of 1.86 µM. It was also found that the combination of fluphenazine and 5-fluorouracil, an antineoplastic drug commonly used for colon cancer therapy, at a concentration of 2 × IC_50_, resulted in less cytotoxicity than fluphenazine alone (Figure 5) [36].

Another study investigated if honeybee venom could synergistically enhance the activity of different CNS drugs, including fluphenazine, in HT-29 colon cancer cells [38]. The authors treated these cells with fluphenazine alone and combined it with increasing concentrations of bee venom for a 48-h treatment. It was found that treatment with fluphenazine + honeybee venom caused a higher reduction in the number of viable cells compared to bee venom alone. Specifically, the combination with fluphenazine +25 µg/mL of bee venom resulted in higher cytotoxicity than both corresponding drugs alone, supporting the use of fluphenazine in adjuvant strategies for colon cancer [38].

### 2.4. Liver Cancer

Faria et al. (2015, [42]) studied the anticancer effects of fluphenazine and other PTZ on the viability of hepatoma cells and found this drug is able to reduce the viability of these cells in a concentration-dependent manner at concentrations between 12.5 and 125 µM. They also found that the addition of different PTZ to cells induced marked morphological changes and increased plasma membrane permeabilization, affecting the mitochondrial transmembrane potential in these cells [42].

A study published by Cheng et al. [43] investigated the effect of different drugs, including the calmodulin antagonist fluphenazine-N-chloroethane, a derivative of fluphenazine, on the Ca^2+^ movement on HA22/VGH hepatoma cells, to determine if they possessed inhibitory effects on Ca^2+^ channels and consequently affect calmodulin activation. The authors concluded that fluphenazine-N-chloroethane, in a concentration range from 2 to 100 µM, did not affect Ca^2+^ levels in these cells [43].

Another study conducted by Hamid et al. [44] evaluated the effect of 117 drugs, including fluphenazine, in the human hepatoma cell line HepG2. Using a single dose screen of 10 µM and using two different cell-based assays (MTT and Alamar blue), the authors found this drug to cause a decrease of more than 55% in cell viability. The authors then determined the EC_50_ of this drug in these cells and found a value of 12.2 and 9.9 µM, for MTT and Alamar assays, respectively [44].

### 2.5. Brain Cancer

One of the first studies of fluphenazine in brain cancer cells dates back to 1994, where Silver et al. studied the anticancer effect of this drug on the human neuroblastoma cell line SK-N-SH [45]. The authors demonstrated that fluphenazine is able to reduce cell viability in a concentration-dependent manner in a wide range of concentrations, with an IC_50_ value of 9.6 µM. They also found that neither treatments with N-methyl-D-aspartic acid (NMDA) nor MK-801 (NMDA receptor antagonist) affected SK-N-SH cell viability, suggesting that the anticancer effect of this drug may not be mediated by dopaminergic receptors [45].

Gil-Ad et al. initiated a series of studies on the anticancer activity of different PTZ in brain-derived tumors a few years ago. In 1998, they demonstrated that some PTZ induced apoptosis in primary mouse brain tissue, human neuroblastoma cells (SK-N-SH), and pheochromocytoma cells (PC12) [46]. At the time, they also reported this effect to be antagonized by some antioxidants, concluding that the development of ROS inside cells could be related to drug-induced cell death [46]. A few years later, the same authors screened several PTZ against rat glioma cells (C6), human neuroblastoma cells (SHSY-5Y), and primary mouse brain tissue to further determine the mechanisms underlying the anticancer effects of this class of drugs [47]. All drugs, including fluphenazine, were able to induce a concentration-dependent decrease in both cell viability and proliferation. Using flow cytometry, the authors also detected an increase of fragmented DNA up to 94% for all drugs in glioma cells. The authors also noted an increase in apoptotic markers in brain-derived cancer cells treated with these drugs, suggesting their potential use in the therapy of brain cancer [47].

De Preter et al. (2009, [48]) performed a meta-mining of neuroblastoma and neuroblast gene expression profiles to find novel therapeutic compounds for neuroblastoma treatment. Neuroblastoma is a childhood cancer that has a very poor outcome, even using multi-approach treatments. The authors found a significant decrease in the viability of five different neuroblastoma cell lines (CLBGA, IMR32, NGP, SKNBE (2c), and SKNSH) treated with five drugs: 17-allylaminogeldanamycin, monorden, fluphenazine, trichostatin, and rapamycin, supporting the anticancer potential of fluphenazine in the treatment of neuroblastoma [48].

A few years later, Cheng et al. [49] studied the effect of fluphenazine and other PTZ on GBM8401 and U87-MG glioblastoma sphere cell lines and found an IC_50_ between 5 and 10 µM for fluphenazine, using an MTT assay. A 72-h treatment also demonstrated this drug is able to reduce cell viability and induce significant cytotoxic effects on GBM8401 cells in a concentration-dependent manner [49].

Using two types of glioblastoma patient-derived mesenchymal (GSC#1) and classical (GSC#9) cells with stem-like properties, Jacobs et al. [50] demonstrated that a 24-h treatment of 20 µM fluphenazine is able to reduce 95% of cell viability and attributed this effect to the inhibition of paracaspase mucosa-associated lymphoid tissue l (MALT1) protease, a protein associated with a worse prognosis of the disease. Using lysates of GSC#9 cells, the authors did not find any effects of the drug in the activation and/or phosphorylation of the proteins AKT, S6, and p70S6K [50]. The authors concluded that inhibition of MALT1 protease induced by fluphenazine increases the number of lysosomes inside cells and impairs autophagic flux, resulting in cell death [50].

### 2.6. Leukemia

Several years ago, Hait and Lee (1985, [51]) evaluated the anticancer effect of fluphenazine against L1210 leukemia cells and found an IC_50_ of 6 µM. The authors also concluded that 14 µM of this drug is able to inhibit calmodulin-activated phosphodiesterase [51].

More recently, fluphenazine and other PTZ were studied by Seredenina et al. (2015, [52]) on a PLB-985 acute leukemia cell line. Using different cell-based assays the authors found that this drug has inhibitory effects on different NADPH oxidases (NOX). NOX 1-5 are involved in the generation of intracellular ROS using intracellular NADPH and play a key role in leukemia [52]. The authors concluded this drug is able to inhibit NOX activity, with IC_50_ values of 13.1, 6.4, 14.5, 7.2, >50, and >50 µM for NOX1, NOX2, NOX3, NOX4, NOX5, and hydrogen peroxide, respectively, using AR assay and 11.6, 9.1, not detected, not detected and 10.2 µM for NOX1, NOX2, NOX3, NOX4, and NOX5, respectively, using the MCLA assay [52].

### 2.7. Oral Cancer

A study published by Cheon et al. (2016, [53]), aimed to increase the sensitivity of highly Halaven (HAL)-resistant cancer cells to chemotherapy. Halaven is the commercial drug name for eribulin mesylate, an antineoplastic drug commonly used for the treatment of resistant or metastatic cancer. To perform the study, the authors used the human oral squamous carcinoma cell line, KB, and its multidrug-resistant parent, KBV20C. The authors found KBV20C cells to be much more resistant to HAL than to other antimitotic drugs. They also found that the concentration needed to treat resistant cells was 500 times more than that necessary for the KB cell line, supporting the need for enhancing the sensitization of KBV20C cells to chemotherapy [53]. To do so, HAL-treated KBV20C cells were co-treated with fluphenazine and it was found that combination treatment with HAL and fluphenazine was able to decrease cell viability and induce apoptosis of resistant cells, via G2 arrest. The mechanism of sensitization seems to occur via alteration of retinoblastoma protein (pRB), pHistone H3, and pH2AX protein levels and by modulation of P-gp activity [53]. Together, these results support that fluphenazine can be used in co-treatments to increase the sensitization of resistant cancer cells and to increase the efficacy of the chemotherapeutic treatment.

Recently, a study published by Kim et al. (2018, [54]) investigated the inhibitory effect of fluphenazine and other drugs on the previously mentioned HAL-resistant KBV20C cancer cells, with focus on its efficacy to increase the sensitization of P-gp-overexpressing resistant KBV20C cancer cells to treatment with HAL. The authors demonstrated that a dose of 10 µM of fluphenazine is able to inhibit P-gP and sensitize HAL-resistant KBV20C cancer cells [54]. Nevertheless, compared to other P-gp inhibitors such as aripiprazole, fluphenazine demonstrates less P-gp inhibitory effect, suggesting it binds to this protein with low specificity. On the other hand, the authors found that the combination of fluphenazine and HAL caused a reduction in cell viability by around 40%, supporting the adjuvant effect of fluphenazine in the treatment of this type of resistant cancer [54].

The same research group delivered another study [55] where they studied the possible effect of the aging-related drugs donepezil and sildenafil citrate on the enhancement of the sensitization of P-gp-overexpressing resistant KBV20C cancer cells to HAL or vincristine. The authors found the combination of HAL and fluphenazine and HAL plus sildenafil citrate to be more effective in the increasing of sensitization of these cells than the combination of HAL plus donepezil [55]. Nevertheless, donepezil was able to inhibit P-gp more effectively than fluphenazine, suggesting that the observed effects of the treatment with HAL plus fluphenazine may involve both cytotoxic and P-gp inhibitory pathways, while the combination of HAL plus donepezil may be a result of only P-gp inhibition by donepezil [55].

Following the previous ideas, a year later, Kim et al. [56] published another study aiming to evaluate the chemosensitizing effect of different histamine receptor antagonists in resistant KBV20C human oral squamous carcinoma cells overexpressing P-gp. The authors found that a combination of low concentrations of loratadine and fluphenazine could successfully sensitize eribulin-resistant KBV20C cells and that co-treatment with 5 µM of fluphenazine with eribulin had a similar effect as a combined treatment of vincristine + fluphenazine, suggesting low doses of this drug can be used in combined approaches with chemotherapeutic drugs to increase chemosensitization, especially in P-gp overexpressing cancer cells [56].

### 2.8. Ovarian Cancer

Using the OVCAR-3 human ovarian cancer cell line, Choi et al. [57] found fluphenazine has a concentration-dependent anticancer effect in these cells, following a 24 h treatment. Mechanistically, the authors attributed the effect of fluphenazine to an epidermal growth factor-induced AKT phosphorylation, rather than by calmodulin inhibition, with an IC_50_ value of 3.84 µM [57]. Pyruvate dehydrogenase kinase 1 (PDK1) was also found to be the target of fluphenazine, suggesting that its inhibition may be involved in PTZ-mediated AKT dephosphorylation and inactivation. The authors also found an increase in the apoptotic cells after fluphenazine treatment, as well as an increase in oligonucleosomal cleavage of genomic DNA and caspase substrate polyadenosine diphosphate ribose, suggesting this drug induces caspase-dependent apoptotic cell death [57].

### 2.9. Melanoma

Besides their promising studies in brain cancer, Gil-Ad et al. (2006, [58]) also evaluated the anticancer effect of fluphenazine and other PTZ on wild-type B16 and MDR B16 mouse melanoma cell lines and found a concentration-dependence effect in the inhibition of cell viability of both these cell lines after a 24 h treatment with fluphenazine, with IC_50_ values of 13.4 and 56.9 µM, respectively [58].

Certain tumors have a huge demand for cholesterol. Targeting cholesterol homeostasis is, therefore, a valuable strategy for cancer combat [59]. More recently, Kuzu et al. evaluated the anticancer effect of different lysosomotropic compounds, including fluphenazine, in a wide range of different melanoma cell lines: UACC903, 1205Lu, 451Lu, 451LuR, WM164M, and C8161.Cl9 and their involvement in the disruption of cancer cell cholesterol homeostasis [59]. The authors studied their effect on intracellular cholesterol levels, cholesterol homeostasis, cellular endocytosis, and signaling cascades. They have found IC_50_ values for fluphenazine in the aforementioned cell lines to be 10.5, 9.5, 24.5, 21.1, 19.1, and 21.7 µM, respectively. They also studied the safety of this drug against FF2441 human fibroblast cells and found the IC_50_ of fluphenazine to be 24.6 µM, indicating the drug is less toxic for normal cells than for melanoma cells [59]. The authors also found that fluphenazine acts by inducing caspase-independent cell death and proved its anticancer activity in a xenograft tumor model, supporting that fluphenazine can effectively disrupt intracellular cholesterol transport and be a potential compound for skin cancer combat [59].

A recent study published by Xia et al. [60] also studied the potential of different PTZ, including fluphenazine, against B16 murine melanoma cells. The authors found an IC_50_ value of 5.1 µM after a treatment of 72 h with this drug [60].

Menilli et al. [61] published a study in 2019 where they evaluated the cytotoxic activity of fluphenazine in combination with UVA light. They used three human tumor cell lines: HeLa (human cervical adenocarcinoma cells), A-431 (skin carcinoma squamous cells), and MSTO-211H (human biphasic mesothelioma cells) and studied the photobiological effect following irradiation treatment in the presence as well as the absence of fluphenazine. They found fluphenazine to be very effective in decreasing the viability of cancer cells in dark conditions (absence of UVA light), with an IC_50_ value of approximately 20 µM. However, when cells were irradiated and co-treated with fluphenazine, the photocytotoxicity was at least two times higher than that after its removal. The authors found that fluphenazine accumulates in lysosomes and distributes in the nucleus and cytoplasm without significant plasma membrane association. After irradiation, it was found that lysosomes and mitochondria inside cells are damaged, resulting in apoptosis, the main mechanism of cell death found in these cells co-treated with fluphenazine and UVA light. It was also found that this combination results in a high generation of ROS, supporting that fluphenazine can be a promising photo-antiproliferative compound [61].

## 3. Discussion on the Future Directions Regarding Fluphenazine Use in Cancer Therapy

Cancer treatment has been evolving in the past decades but the number of effective methods of treatment is still very low, resulting in an increase in diagnosed patients with this disease over the years. Drug repurposing is a strategy that has been studied for several diseases, including cancer, that tries to find new uses for existing drugs. This is advantageous over the development of new drugs as it allows to save money and time since these drugs are already well-defined in terms of pharmacological and toxicological risks, making them more accessible for clinical trials. Several studies have supported the different roles of antipsychotic drugs such as fluphenazine and other PTZ in proteins and signaling pathways related to tumorigenesis, in different types of cancer [11,12,13]. The previously mentioned studies analyzed the anticancer activity of fluphenazine and a vast majority have confirmed the potential of this drug as an anticancer agent capable of decreasing the viability of different cancer cells, in concentrations of micromolar. Collectively, these studies have demonstrated that fluphenazine causes DNA fragmentation, induces cell cycle arrest, activates different caspases, regulates ROS generation and ATP levels, induces apoptosis, and decreases cell migration and invasion. Some studies also demonstrate that fluphenazine is able to act as a chemosensitizer when combined with other drugs, UVA light, or other compounds. Although the exact mechanism of action of fluphenazine in cancer is not yet fully established because more accurate studies are still necessary, these preliminary findings demonstrate that this drug acts on cell cycle, proliferation, and apoptosis; it can induce ROS production which affects the mitochondrial potential and causes DNA damage, that results in cell cycle arrest and DNA fragmentation that ultimately leads to cell death by apoptosis, increasing the expression of Bax and caspases. It is also possible that the cell cycle arrest induced by fluphenazine causes a decrease in the mitochondrial potential, which in turn leads to DNA fragmentation and apoptosis. It can also induce lysosomal dysfunction as well as a decrease in ABCB1 and P-gp binding, increasing the sensitization of drug-resistant cells [14]. It can also act on the Akt/Wnt pathway, an important signaling mechanism that regulates cell metabolism, cell survival, apoptosis, etc., which is known to be modulated by D2 receptor antagonists [14]. The Wnt signaling pathway is complex and its regulation occurs at multiple levels as it affects the cytoplasmic regulation of phosphorylation of β-catenin, nuclear localization of β-catenin, and transcriptional activation, regulating cell proliferation and therefore having an important role in cancer [62]. On the other side, the Akt cascade is another important pathway in cancer as, it can regulate different proteins such as FoxO1, a regulator of the cell cycle, BAD, a pro-survival factor, and mTOR, a key regulator of cell growth and metabolism [63]. Taken together, the previously mentioned promising in vitro results using both human and animal cancer cells support further in vivo studies and clinical trials.

Different studies have already reported the in vivo efficacy of fluphenazine against different types of cancer. Using mice bearing TNBC xenografts, Goyette et al. found a reduction in tumor size and number of metastasis in mice treated with fluphenazine [34]. Another study investigated the potential of fluphenazine for the treatment of TNBC with brain metastasis and found that BALB/c mice treated with fluphenazine showed a brain/plasma drug concentration ratio above 25 after 24 h treatment, demonstrating the ability of this drug to cross the blood–brain barrier [35]. Using a TNBC subcutaneous xenograft mouse model and a mouse brain metastasis model, the authors found that fluphenazine is able to decrease tumor growth as well as inhibit 85% of brain metastasis. This drug demonstrated a strong effect in the inhibition of spontaneous lung metastasis, without causing undesired side effects [35]. Another study, using melanoma xenografts, also showed that fluphenazine treatment was effective in inhibiting tumor growth, in a mechanism related to disruption of intracellular cholesterol transport [59]. In ovarian cancer, mouse xenografts treated with fluphenazine presented a significant reduction in tumor size, in a short-period treatment. For longer periods, fluphenazine treatment resulted in animal death, suggesting that side effects related to CNS may impair fluphenazine use in cancer [57]. Kuzu et al. suggest that these problems may be overcome using a nanoparticle formulation that can help to decrease the passage of the drug over the blood–brain barrier while increasing the permeation into the tumor [59]. Taken together, these in vivo results suggest that fluphenazine may be used for cancer therapy, although with care to minimize the side effects associated with the use of this drug. Furthermore, other side effects already reported for its original indication should also be accounted for when using fluphenazine for novel indications; this drug has an adverse effect profile similar to other antipsychotics, and the most common side effects include sedation, dry mouth, dry eyes, blurred vision, constipation, orthostasis, dizziness, hypotension, and urinary retention [64]. Other less common adverse effects can include rebound tachycardia, urinary retention, and weight gain as well as akathisia, parkinsonian features such as resting tremor and shuffling gait, acute dystonic reactions, oculogyric crises, opisthotonos, and tardive dyskinesia, that can be manageable with medications such as benztropine or sodium benzoate [64]. Rare side effects include neuroleptic malignant syndrome, liver function abnormalities and jaundice, seizures, and agranulocytosis. It can also increase the risk of cerebrovascular events. Some reports also describe allergic reactions related to the use of fluphenazine [64]. Therefore, the indication of fluphenazine in most cases seems to induce side effects that are similar to most medications and compatible with clinical use. Nevertheless, clinicians should exercise caution using this drug and must be aware of the eventual development of severe side effects that can occur upon administration of fluphenazine, especially in patients with hepatobiliary disease or hepatic insufficiency and in elderly with psychosis associated with major neurocognitive disorder and other dementia-related illnesses [64]. The risks and benefits of prescribing fluphenazine for cancer treatment should, therefore, be weighed against possible side effects guiding use.

## 4. Conclusions

This review provides solid support of the anticancer activity of fluphenazine against different types of cancer, both in vitro and in vivo studies. It has been demonstrated that this drug is able to fragment DNA, induce cell cycle arrest, induce apoptosis and decrease the ability of cancer cells to migrate and invade. The mechanism of action of fluphenazine in cancer is not fully established but it appears to induce ROS production, affecting the mitochondrial potential and causing DNA damage, which results in cell cycle arrest and DNA fragmentation, leading to cell death by apoptosis. These preliminary results indicate fluphenazine may target the Akt and Wnt signaling pathways, that in turn can affect the expression of important downstream proteins such as β-catenin, FoxO1, BAD, and mTOR, ultimately regulating molecular processes related to cell metabolism, cell survival, apoptosis, etc. The in vivo results demonstrate this drug is a potent inhibitor of tumor growth and can decrease the metastasizing of cancer cells, although with some side effects associated. The use of fluphenazine in cancer therapy seems to be promising but further studies are needed to define the mechanisms of action of this drug in cancer, to help find and design novel derivatives with enhanced activity and decreased associated side effects. The potential of fluphenazine as an anticancer compound is further demonstrated by the ongoing clinical trials.

## Data Availability

Not applicable.
